# The Validity of Self-Initiated, Event-Driven Infectious Disease Reporting in General Population Cohorts

**DOI:** 10.1371/journal.pone.0061644

**Published:** 2013-04-17

**Authors:** Hanna Merk, Sharon Kühlmann-Berenzon, Christin Bexelius, Sven Sandin, Jan-Eric Litton, Annika Linde, Olof Nyrén

**Affiliations:** 1 Department of Medical Epidemiology and Biostatistics, Karolinska Institutet, Stockholm, Sweden; 2 Department of Analysis and Prevention, Swedish Institute for Communicable Disease Control, Solna, Sweden; 3 OptumInsight, Stockholm, Sweden; 4 Department of Medicine, Vanderbilt University School of Medicine, Nashville, Tennessee, United States of America; Harvard School of Public Health, United States of America

## Abstract

**Background:**

The 2009/2010 pandemic influenza highlighted the need for valid and timely incidence data. In 2007 we started the development of a passive surveillance scheme based on passive follow-up of representative general population cohorts. Cohort members are asked to spontaneously report all instances of colds and fevers as soon as they occur for up to 9 months. Suspecting that compliance might be poor, we aimed to assess the validity of self-initiated, event-driven outcome reporting over long periods.

**Methods:**

During two 8 week periods in 2008 and 2009, 2376 and 2514 cohort members in Stockholm County were sent one-week recall questionnaires, which served as reference method.

**Results:**

The questionnaires were completed by 88% and 86% of the cohort members. Whilst the false positive proportion (1–specificity) in the reporting was low (upper bound of the 95% confidence interval [CI] ≤2% in each season), the false negative proportion (failure to report, 1–sensitivity) was considerable (60% [95% CI 52%–67%] in each season). Still, the resulting epidemic curves for influenza-like illness compared well with those from existing General Practitioner-based sentinel surveillance in terms of shape, timing of peak, and year-to-year variation. This suggested that the error was fairly constant.

**Conclusions:**

Passive long-term surveillance through self-initiated, event-driven outcome reporting underestimates incidence rates of common upper respiratory tract infections. However, because underreporting appears predictable, simple corrections could potentially restore validity.

## Introduction

Infectious disease surveillance typically relies on reporting from health care [1], [2]. Such reporting has limitations when it comes to continued monitoring of epidemics. The threshold for health care consultations may vary over time, and international differences in health care structure, consultation behaviour and reporting lead to international differences that are difficult to interpret [3]. To fully appreciate the societal consequences of infectious diseases, surveillance should cover the entire disease spectrum in the population. This requires data collection directly among the public. To provide valid incidence data in real-time directly from representative lay people, we started to develop a population-based surveillance scheme in Stockholm County, Sweden, in 2007 [4]. The system is based on yearly recruitment of representative general population cohorts, whose members are asked to spontaneously report all new events of colds and fevers as soon as they occur during a follow-up period of up to 9 months including the influenza season. This passive surveillance yields incidence data in close to real-time and avoids irritating repetitive queries. Automated telephone and web technologies allow simultaneous reporting from several hundred participants at comparatively low cost for the investigator, thus making large-scale efforts possible.

Suspecting limited compliance with this event-driven self-reporting (henceforth referred to as “event-driven reporting”), we set out to assess its validity when used in surveillance of influenza during the seasons 2007/2008 and 2008/2009. Our main focus was validation with one-week recall questionnaires, but we also compared the event-driven reporting to an end-of-follow-up questionnaire and to the routine sentinel surveillance.

## Methods

### Ethics Statement

The studies were reviewed and approved by the Stockholm Regional Research Ethics Review Board (2007/952-31, 2007/1599-32, 2008/1227-32). Actively registering in the surveillance system and returning a questionnaire was considered as giving informed consent.

### The Population-based Surveillance System

Starting in 2007 residents of Stockholm County (0–95 years of age) have annually been randomly selected from the Swedish population register to receive mailed invitation letters simultaneously before start of the influenza season, asking them to register in the surveillance system [4]. For children, guardians have been recruited as proxy responders. The event-driven reporting of colds and fevers has been done via a secure website or a toll-free telephone service with interactive voice response. Regardless of communication technology, the cohort members have been required, as part of their event-driven report, to answer a brief tree-structured symptom questionnaire (Table S1). The questionnaire has consisted of 12–14 lay language, multiple choice questions, which have mainly probed into symptoms in the case definition for influenza-like illness (ILI) proposed by the European Centre for Disease Prevention and Control (ECDC) [5]. Symptom algorithms adapted from the ECDC case definitions [5] have determined if the disease episodes classified as acute upper respiratory tract infection (AURTI), ILI or other (Table S1). As part of the evaluation program, invitees from 2007/2008 who expressed an interest in future studies were re-invited to participate during 2008/2009, in addition to the randomly selected new invitees. Monthly reminders have normally been sent via regular mail or e-mail, but in the 2007/2008 season they were sent out on only two occasions. The follow-up periods have typically covered 33 weeks from mid-September through May, but in the 2008/2009 season the surveillance did not start until late December. Since people may be more inclined to register for the surveillance when they are ill, we have routinely discarded the first two weeks of follow-up to avoid selection bias. The cohorts in the 2007/2008 and 2008/2009 seasons consisted of 3447 and 3954 individuals, respectively. Of the latter, 1604 (41%) also participated in the 2007/2008 cohort.

### Validation Study

The validation effort took place during ongoing surveillance in two succeeding cohorts between January 14 and March 9, 2008, and between January 26 and March 22, 2009. We used one-week recall questionnaires as reference method for these time periods. For each individual, the one-week validation periods were randomly selected; in 2008 there were 2–3 validation weeks per participant and in 2009 2 validation weeks per participant. The participants were unaware of the timing of these weeks. The restriction in time was applied primarily to contain costs. Although not a perfect gold standard, the one-week recall questionnaire was used as reference method. In other fields of epidemiology, notably nutritional epidemiology, this method has attained acceptable validity in varying populations, children and elderly excepted [6]. While the retrospective reports might have been susceptible to recall errors, such reporting caused minimal interference with the investigated prospective reporting. Moreover, the errors associated with the two reporting methods were deemed to be reasonably uncorrelated. The questionnaires were distributed via e-mail when possible, otherwise by regular mail, and consisted of two questions: 1. Did you have a cold or fever last week [exact dates specified]? 2. If yes, did it start last week? (The validation week was always Monday-Sunday the preceding week.) All cohort members in both periods received questionnaires, except for approximately 1000 randomly selected cohort members per period who were left undisturbed. The latter groups enabled us to assess if the validation affected the event-driven reporting (reactivity).

**Figure 1:**
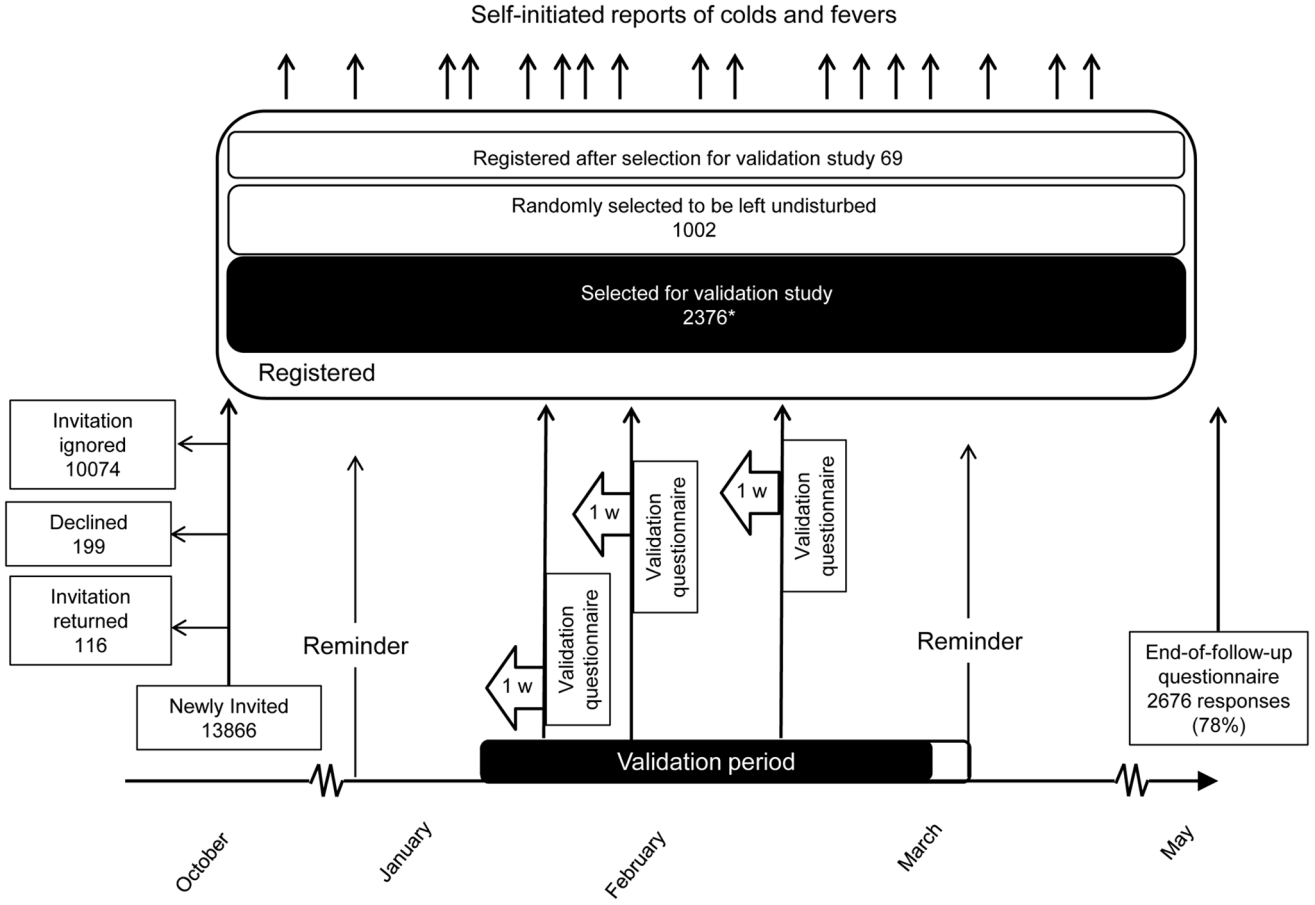
The population-based surveillance system and validation study during the influenza season 2007/2008. Following an invitation to a randomly selected population sample at ages 0–95 years around October 1^st^ 2007, registered cohort members were followed passively, relying on self-initiated, event-driven reporting of all colds and fevers, until May 30^th^ 2008. Reminders were sent out at Christmas and Easter, and an end-of-follow-up questionnaire around May 30^th^. The validation study consisted of 2–3 randomly selected validation weeks per participant between January 14 and March 9 2008, with 1-week recall questionnaires. *Participation rate 2082/2376 (88%); another 43 were excluded due to uninterpretable reports or interference in the last week by the Easter reminder. The Easter reminder arrived when it was still possible to submit a legitimate event-driven report.

**Figure 2:**
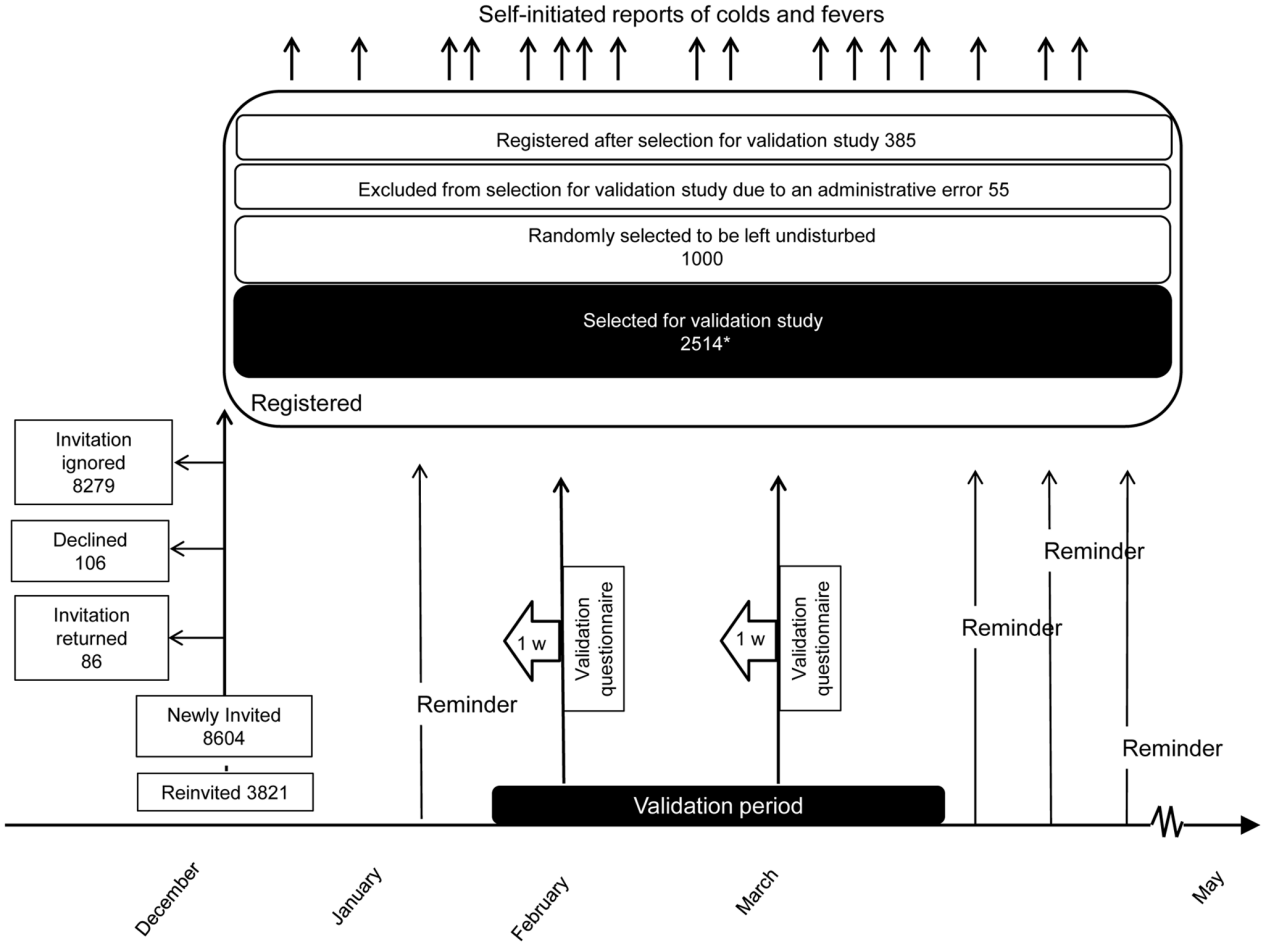
The population-based surveillance system and validation study during the influenza season 2008/2009. The invitation was delayed until late December 2008. In addition to the random population sample, invitees from the previous season who expressed an interest in participating in future studies were re-invited. After start of follow-up, participants received reminders every fourth week. There was no end-of-follow-up questionnaire. Of all registered participants 1604 were re-entered from the previous season and 2350 were new participants. The validation study consisted of 2 randomly selected validation weeks per participant between January 26 and March 22 2009, with 1-week recall questionnaires. *Participation rate 2155/2514 (86%); another 21 were excluded since we did not consider the first two weeks after entry into the surveillance cohort, cohort members whose only validation week fell on any of these weeks were excluded. Uninterpretable reports were also excluded. Of the 2514 selected 1062 had re-entered and 1452 were new participants.

At the end of follow-up in May 2008 we also distributed a postal questionnaire asking all who had been invited about the number of colds and fever episodes experienced between October 1 2007 and May 25 2008. The passage of cohort members through the validation studies in 2008 and 2009 is illustrated in Figures 1 and 2.

### Statistical Aspects

#### Validity analysis

We compared the reported absence or presence of fever or cold in the one-week recall reference method, with the absence or presence of an event-driven report consistent with fever or AURTI with onset in the specified week. The main result measures were: *false negative proportion* (1–sensitivity, i.e. failure to report disease episodes ascertained with the reference method), *false positive proportion* (1–specificity, i.e. reports of episodes not confirmed by the reference method), *positive predictive value* (the probability of disease according to the reference method given an event-driven report of disease), *negative predictive value* (the probability of being disease-free according to the reference method given the absence of an event-driven report). Confidence intervals (CI) were calculated with the exact binomial method.

To avoid the problem of dependency of reports within the same individual, only the first validation week of each participant was included in the main analysis. A supplementary analysis included all eligible validation weeks, and a second supplementary analysis included event-driven reports also in the *two* weeks preceding the validation week, to allow for possible telescoping bias, i.e. incorrect inclusion, in the reference measurements, of infections that started *before* the retrospective one-week time window that was to be recalled [7]. Since the two compared methods differed slightly in regard to the definition of disease events that were to be reported, we investigated, in a third supplementary analysis, the validity after redefining a positive event-driven report as any report of one or more symptoms regardless of type of symptom.

The validity measures were calculated separately for each season, by age groups and by validation week. We compared the false negative and false positive proportions in the first validation week with those in the second and third, and tested the ratio between the proportions with a Wald test on the log scale [8]. Based on the prospective event-driven reporting, incidence rates with 95% CI were calculated for the entire 2007/2008 season, and for 2008/2009 by dividing the number of reported fever/AURTI episodes by the total person-time accrued. Incidence rates among cohort members selected for the validation effort were compared with the corresponding rates among the cohort members who were left undisturbed. We used the Wald test under the assumption of Poisson distribution of the rates to test if the rate ratio was significantly different from unity [9].

We further compared each cohort member’s total number of disease episodes based on the event-driven reports in the 2007/2008 season to the number retrospectively reported in the end-of-follow-up questionnaire using the Wilcoxon matched pairs signed rank sum test [10].

#### Risk indicators for failure to report

Logistic regression modelling identified factors associated with a failure to report AURTI or fever occurrence when the response in the reference questionnaire indicated onset of cold or fever (henceforth referred to as “false negatives”). The analysis was restricted to the 2007/2008 cohort for which we had information on the individuals’ education level, marital status, household size, and household income, collected from registers held by Statistics Sweden. The model included gender, age (0–14; 15–39; 40–64; ≥65 years), length of education (for children, the reporting guardian’s length of education) (≤9; 10–14; ≥15 years; missing data), household size (1; 2; 3; 4; ≥5 persons; missing data), household income in 2006 (≤226,810; 226,811–340,466; 340,467–473,903; ≥473,904 SEK; missing data), time from invitation to registration (≤2 weeks; >2 weeks), mode of registration (Internet; telephone), event-driven disease report within 24 hours of registration (yes; no), and calendar time expressed as week of the validation period (1; 2; 3; 4; 5; 6; 7). All the individual’s validation weeks with cold or fever onset according to the reference were included.

The model was fitted as a generalized linear model (GLM). Random effects and GEE to account for dependency between reports from the same individual, were not feasible to fit. In order to determine if much error was incurred by not including the dependency between reports, we compared the estimated coefficients and standard errors of the model containing only main effects but with calendar time as continuous variable, with and without random effects, and also calculated the significance of the within-person clustering. Only minute differences were found between the estimates of the coefficients, and the clustering was not significant (rho = 0.07, p = 0.41). In the final model calendar time was treated as a categorical variable since closer inspection showed that a linear relationship could not be assumed. All two-way interactions were explored in a forward stepwise manner. The most significant candidate interaction was kept if the likelihood ratio test between the current and last model had a p<0.05. From the model we obtained odds ratios and 95% Wald CI.

The ordinal variables that were significant in the GLM model were additionally tested for departure from linear trend by comparing the model with dummy variables and the same model with the variable recoded as a quantitative score. The following scores were used: age as group medians (5; 29; 52; 71 years) and education as ordered categories (1; 2; 3). Where the likelihood ratio test had a p>0.05, we interpreted the odds ratio from the quantitative score as the increase or decrease of the risk for a false negative report for every increase in the score [11].

#### Correspondence with routine sentinel surveillance of influenza

We compared the epidemic curves for ILI generated from the event-driven reporting during the first 5 months of 2008 and 2009 with curves from the Swedish routine sentinel influenza surveillance based on General Practitioners using cross-correlation with varying lag times [12].

#### Adjustment for misclassification

The true number of ILI cases was estimated using the following formula
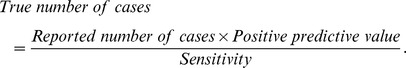



The sensitivity and positive predictive value for each season and in strata of age and sex in combination were calculated and applied to our formula. The true number of cases was added together for each week and divided by the total number of participants for each week, resulting in an adjusted weekly incidence proportion of ILI.

STATA 12 (StataCorp; USA) was used in all analyses.

## Results

Table 1 shows characteristics of invited and participating individuals in the validation efforts. A total of 2039 and 2134 cohort members participated in 2008 and 2009, respectively. Thus, the participation rates were 86% and 85%. Of these, 934 (46%) and 910 (43%) submitted at least one disease report, and the total number of reports was 1365 and 1085. At least one interpretable cold or fever onset was reported in the validation questionnaires by 371 and 291 participants in 2008 and 2009, respectively. The mean age was somewhat higher and the female predominance slightly more pronounced in 2009 than in 2008. Among sociodemographic variables (available only for the 2008 cohort), it was seen that 63% of the participants had 10 years or more of education, and 27% lived in single-person households. Since the 2009 cohort consisted of a mix of participants who were newly entered and participants who were re-entered from the previous cohort, we examined recorded characteristics among validation participants in these two subsets (Table S2). Although there was a greater female predominance (61% vs. 57%, p = 0.046) and a higher proportion who reported at least one disease episode (45% vs. 41%, p = 0.051) among the re-entered, the differences were generally small.

**Table 1:** Characteristics of individuals selected for and included in the validation of the event-driven reporting in the population-based disease surveillance in Stockholm County 2008 and 2009.

		2008						2009					
		Selected (n = 2376)			Included in analysis^a^ (n = 2039)			Selected (n = 2514)			Included in analysis^a^ (n = 2134)		
		No.	%	SD	No.	%	SD	No.	%	SD	No.	%	SD
Men		1040	44		888	44		1074	43		872	41	
Mean age, years		42		24	43		24	46		24	48		24
Age groups, years	≤14	464	20		374	18		413	16		313	15	
	15–39	562	24		454	22		530	21		406	19	
	40–64	895	38		782	38		924	37		815	38	
	65–95	455	19		429	21		647	26		600	28	
Child ≤15 years		495	21		397	19		448	18		339	16	
Educational level, years	≥15	564	24		492	24							
	10–14	921	39		799	39							
	≤9	180	8		150	7							
	Missing^b^	711	30		598	29							
Marital status	Single	1075	45		894	44							
	Married^c^	924	39		813	40							
	Divorced	233	10		206	10							
	Widowed	96	4		91	4							
	Missing	48	2		35	2							
Household size	1	634	27		557	27							
	2	605	25		550	27							
	3–4	866	36		713	35							
	≥5	229	10		186	9							
	Missing	42	2		33	2							
Subjects with at least one event-report		1042	44		934	46		993	39		910	43	
Total number of complete event-reports		1486			1365			1183			1085		
Subjects with at least one interpretable cold or fever onset in the validation questionnaires		371			371			291			291		

a Included in the analysis were selected individuals who returned at least one validation questionnaire with interpretable answers.

b The “Missing” category included children who had not yet finished school, selected children n = 495, included children n = 397.

c The “Married” category included individuals in registered partnerships.

### Validity

While the false positive proportion was no more than 1% in both seasons (upper bound of the 95% CI ≤2%), the false negative proportion (failure to report) was 60% (95% CI 52%–67%) in the first season and 60% (95% CI 52%–67%) in the second (Table 2). The lowest false negative proportion was observed for children, but with few exceptions the variation between age groups was small. Using all observations (i.e. more than one validation week per participant) yielded similar results; the false positive proportion was 1% in both seasons, while the false negative proportion was 66% (95% CI 61%–70%) and 60% (95% CI 54%–65%) in the first and second season, respectively. The negative predictive values (94% for the first and second season) were higher than the positive predictive values (79% and 88%, Table 2). A supplementary analysis with positive event-driven reports redefined as any report of one or more symptoms, regardless of type of symptom, yielded results that were only trivially different from the results of the main analysis (data not shown). The inclusion of re-entered cohort members in the second season did not have any major impact on the results; among re-entered and newly entered, the false negative proportions were 61% and 59%, respectively, and the false positive proportions 0% and 1% (Table S3).

**Table 2:** Observed false negative proportion, false positive proportion and predictive values in the 2008 and 2009 validation divided by age group and overall.

	Age group	2008		2009	
		%	95% CI	%	95% CI
False negative proportion	0–14	36/64 (56%)	(43–69)	28/51 (55%)	(40–69)
	15–39	28/46 (61%)	(45–75)	19/33 (58%)	(39–75)
	40–64	33/53 (62%)	(48–75)	33/57 (58%)	(44–71)
	≥65	11/18 (61%)	(36–83)	23/32 (72%)	(53–86)
	Total	108/181 (60%)	(52–67)	103/173 (60%)	(52–67)
False positive proportion	0–14	4/275 (1%)	(0–4)	3/217 (1%)	(0–4)
	15–39	5/350 (1%)	(0–3)	3/292 (1%)	(0–3)
	40–64	10/674 (1%)	(1–3)	2/638 (0%)	(0–1)
	≥65	0/375 (0%)	(0–1)	2/477 (0%)	(0–2)
	Total	19/1674 (1%)	(1–2)	10/1624 (1%)	(0–1)
Positive predictive value	0–14	28/32 (88%)	(71–96)	23/26 (88%)	(70–98)
	15–39	18/23 (78%)	(56–93)	14/17 (82%)	(57–96)
	40–64	20/30 (67%)	(47–83)	24/26 (92%)	(75–99)
	≥65	7/7 (100%)	(59–100)	9/11 (82%)	(48–98)
	Total	73/92 (79%)	(70–87)	70/80 (88%)	(78–94)
Negative predictive value	0–14	271/307 (88%)	(84–92)	214/242 (88%)	(84–92)
	15–39	345/373 (92%)	(89–95)	289/308 (94%)	(91–96)
	40–64	664/697 (95%)	(93–97)	636/669 (95%)	(93–97)
	≥65	375/386 (97%)	(95–99)	475/498 (95%)	(93–97)
	Total	1655/1763 (94%)	(93–95)	1614/1717 (94%)	(93–95)

### Allowing for Possible Telescoping Bias in the Reference Measures

Extending the time window for the event-driven reporting to 3 weeks, i.e. adding to the assigned validation week the 2 preceding weeks of event-driven reporting, reduced the false negative proportion to 51% (95% CI 44%–59%) in 2008 and to 48% (95% CI 39%–56%) in 2009, with only minor increases in the false positive proportions (5% in 2008 and 7% in 2009).

### Validity Over Time

Validity did not change significantly with time. In 2008 the false negative proportion increased from 60% (95% CI 52%–67%) in the first validation week to 69% (95% CI 61%–76%) in the second and 75% (95% CI 63%–85%) in the third (p = 0.98 for the third versus the first week). The false positive proportions in the corresponding weeks were 1% (95% CI 1%–2%), 1% (95% CI 0%–1%) and 0% (95% CI 0%–1%). In 2009, when reminders were more frequent, the false negative proportions also remained unchanged (first validation week 60%, [95% CI 52%–67%]; second validation week 60%, [95% CI 51%–68%]). The false positive proportions were 1% (95% CI 0%–1%) in both weeks. Among re-entered cohort members, the false negative proportion went from 61% to 56% and the false positive proportion from 0% to 1%. The corresponding figures for newly entered cohort members were 59% to 63% and 1% to 0% (data not shown).

### Comparison with Retrospective Reporting at the End of the Season

In May 2008, 2676 (78%) cohort members answered the end-of-follow-up questionnaire, which indicated that the incidence of cold or fever had been 41 per 1000 person-weeks. Prospective event-driven report data for the same season showed an incidence of AURTI or fever of 24 per 1000 person-weeks (95% CI 23–25) for cohort members selected for the validation and 22 per 1000 person-weeks (95% CI 20–24) for those not selected (p = 0.07). The number of prospective event-driven reports per individual differed significantly from the number of retrospective end-of-follow-up reports (p<0.0001), Table 3. In 2009 the incidence of AURTI or fever was 24 per 1000 person-weeks for those selected for validation and 20 per 1000 person-weeks for those not selected (p = 0.0001).

**Table 3:** Number of persons by number of illness episodes in the end-of-follow-up questionnaire and number of event-driven surveillance reports during the entire 2007/2008 season.

		Event-driven surveillance reports of illness, No.					
		0	1	2	3	4	≥5
	0	727	81	7	0	0	0
	1	425	292	28	0	0	0
End-of-follow-up questionnaire illness episodes, No.	2	225	200	105	15	3	0
	3	84	77	49	14	2	0
	4	18	31	24	13	8	2
	≥5	22	16	15	6	12	9

Wilcoxon matched pairs signed rank sum test p<0.0001.

### Risk Indicators for Failure to Report

The final logistic model of 2008 data included two significant interactions (p<0.05); (1) between age and mode of registration, and (2) between gender and time from invitation to registration. The model revealed late-responding men, elderly who registered via the internet and low education as independent risk indicators for failure to report. Children (for whom a guardian did the reporting) who were registered via the internet had lower risk for false negatives than the reference consisting of participants aged 40–64 years who registered via the telephone. In general, among those registered via the internet, there was a linear trend (odds ratio 1.03 [95% CI 1.01–1.05]) for increasing risk with increasing age category (Table 4). Cohort members who reported disease within 24 hours after registration showed no evidence of being more negligent than the others. Although the risk for false negatives increased somewhat with time, the point estimates did not reach statistical significance and trend was therefore not tested.

**Table 4:** Multivariable logistic regression modelling of the association between background factors and risk of false negative reporting (i.e. no report through the population-based, event-driven surveillance system when the reference method – one-week recall questionnaires – signals onset of disease) in 2008, n = 396.

		OR	95% CI
Validation week	1	1.00	
	2	0.59	(0.25–1.43)
	3	0.70	(0.28–1.75)
	4	1.13	(0.45–2.82)
	5	1.01	(0.40–2.57)
	6	1.32	(0.56–3.10)
	7	1.21	(0.46–3.18)
Gender, and Time from invitation to registration	Women ≤2 weeks	0.95	(0.45–2.01)
	Women >2 weeks	1.00	
	Men ≤2 weeks	1.53	(0.68–3.42)
	Men >2 weeks	6.37	(2.05–19.82)
Age group, years, and Mode of registration	0–14 Telephone	1.07	(0.36–3.19)
	15–39 Telephone	1.44	(0.46–4.52)
	40–64 Telephone	1.00	
	≥65 Telephone	1.24	(0.36–4.29)
	0–14 Internet	0.38	(0.16–0.94)
	15–39 Internet	0.68	(0.27–1.67)
	40–64 Internet	1.33	(0.55–3.21)
	≥65 Internet	11.58	(1.17–114.24)
Educational level, years^a^	≤9	1.00	
	10–14	0.24	(0.08–0.69)
	≥15	0.25	(0.08–0.74)
	Missing	0.24	(0.04–1.58)
Household size	1	1.00	
	2	0.59	(0.25–1.41)
	3	1.76	(0.69–4.54)
	4	1.26	(0.47–3.36)
	≥5	1.48	(0.50–4.39)
	Missing	0.69	(0.03–15.71)
Household income group^b^	Low & Middle/low	1.00	
	Middle	1.81	(0.69–4.73)
	Middle/high	1.34	(0.53–3.39)
	High	0.80	(0.35–1.80)
	Missing	4.72	(0.45–49.27)
Event-driven disease report within 24 hours of registration	No	1.00	
	Yes	1.03	(0.60–1.78)

a Education is the guardians’ highest education if child.

b Low & Middle/low≤226,810; Middle = 226,811–340,466; Middle/high = 340,467–473,903; High≥473,904 in SEK in 2006.

### Correspondence with Sentinel Surveillance

**Figure 3:**
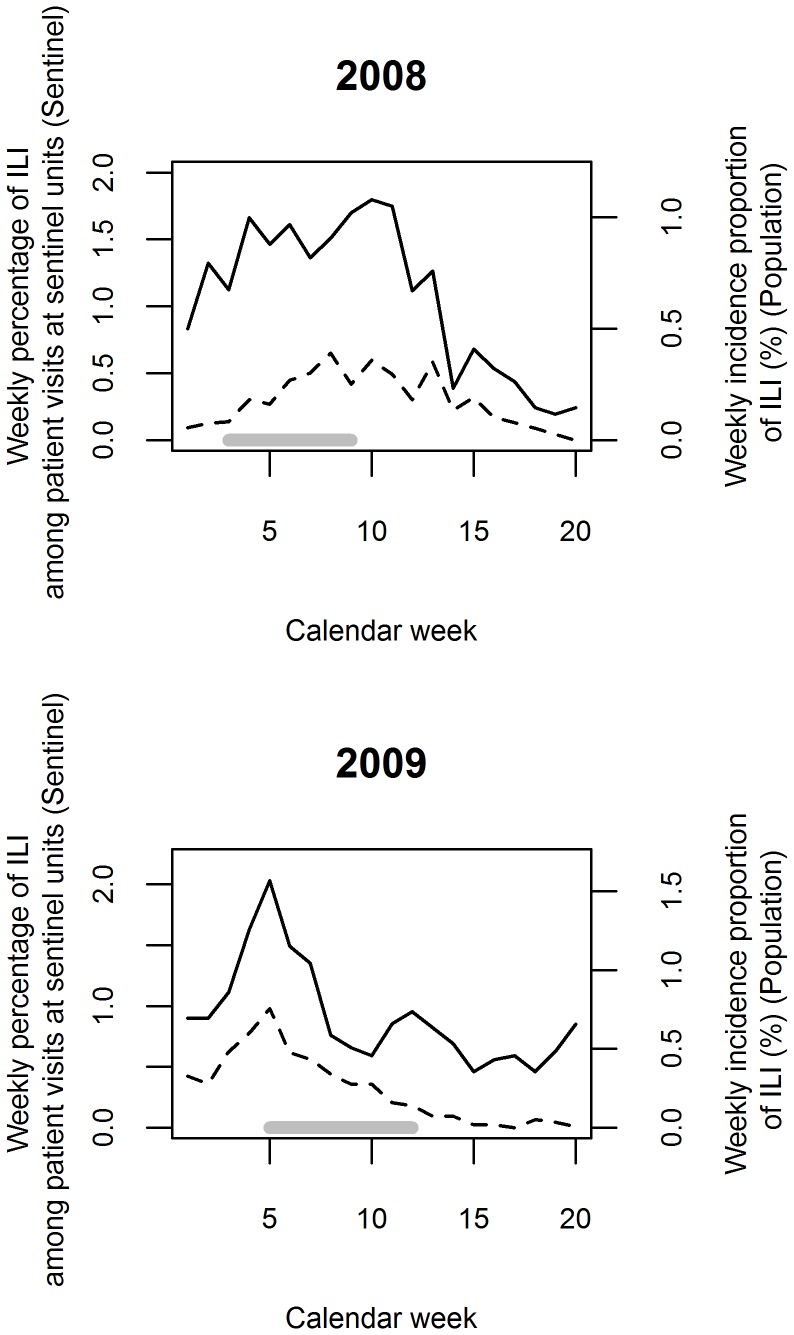
Epidemic curves for influenza-like illness (ILI). The curves have been derived from the existing sentinel surveillance in Sweden (dashed line) and the passive follow-up with self-initiated, event-driven outcome reporting in population-based surveillance cohorts (solid line). The upper graph represents 2008, the lower one 2009. The solid grey horizontal lines indicate start and end of the validation efforts included in the analysis. Please note that sentinel and population-based surveillance measured different aspects of ILI occurrence. Attempts by ECDC to transform Swedish sentinel data to approximate weekly incidence proportions resulted in estimates almost one order of magnitude lower than the observed incidence proportions in the population-based surveillance cohort.

ILI epidemic curves compared well with routine Swedish sentinel surveillance curves in terms of shape, timing of the peak, and year-to-year variation (Figure 3). Cross-correlation analysis showed that maximum correlation was attained when no lag time was applied. In 2008 the cross-correlation was r = 0.76 and in 2009, r = 0.88 (p<0.05 for both).

### Adjustment for Misclassification

**Figure 4:**
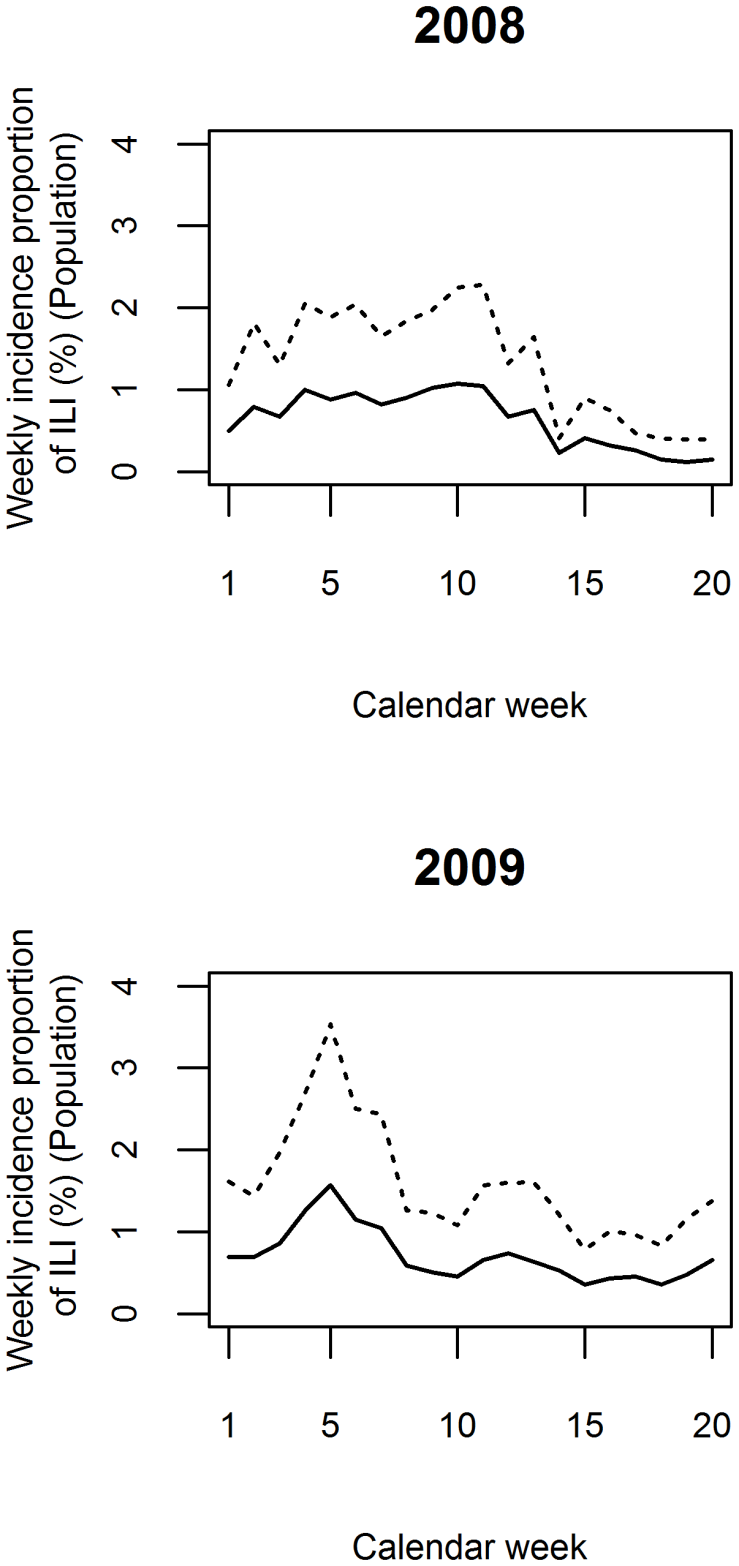
Epidemic curves for influenza-like illness (ILI). The curves are derived from the passive follow-up with self-initiated, event-driven outcome reporting in population-based surveillance cohorts (solid line) and adjusted for imperfect sensitivity (dashed line). The upper graph represents 2008, the lower one 2009.

Using the observed season- and stratum-specific sensitivity and positive predictive values, we corrected the weekly incidence proportions (Figure 4). The shapes of the adjusted epidemic curves for 2008 and 2009 were similar to the unadjusted ones, but the weekly incidence proportions were higher peaking at 2.3% in 2008 and 3.5% in 2009.

## Discussion

Although the interpretation of discrepancies in relation to an imperfect reference is uncertain, two different reference standards both indicated that passive follow-up for common respiratory infections relying on event-driven reporting suffers from important under-reporting, which – in the setting at hand – seemed to be remarkably constant. There were very few false positive reports, the overall positive predictive values were around 80% or above, and the negative predictive values were greater than 90%.

Strengths of our validation study include the high response rate, comparisons with three different alternative sources of information, large representative undisturbed subsamples enabling us to assess whether the validation study affected the event-driven reporting, and the repeated validation during two seasons.

An important weakness is the lack of a solid gold standard. To compare retrospectively and prospectively collected data is problematic [13]. Telescoping bias affecting the reference method is one concern that received some support in our data. Like us, Wheeler et al found a much higher incidence of intestinal infections when comparing a retrospective estimate to a prospective one within the same study [14]. However, if reactivity (i.e. interference with measurements under evaluation) is to be avoided, there are few realistic alternatives to retrospective self-reporting. Since no measurement is without error, the focus ought to be on whether or not the errors of the investigated measurement and the reference measurement are independent. Our reference standard recall questionnaires were simple to fill out and administered in a way that was, in practice, unpredictable for the participants. However, as we accepted event-driven reports for up to 7 days after disease onset, participants could submit a legitimate event-driven report concerning the validation week after the one-week recall questionnaire had arrived. Hence, the reference measurements might theoretically have interfered with the event-driven reporting, but our data provided support for no more than a weak impact. Therefore, the evaluated reporting was only marginally affected by the standard reference method, and the errors were expected to be reasonably uncorrelated.

Previous studies relying on data collection among the public confirm the particularly poor cooperation by men [15]–[17] and people with a low level of education [18]–[20] observed by us. The effects of male gender and old age on drop-out rate in a longitudinal study have previously been found to be modified by subjective health [21]. In our study, the infections per se might have influenced the ability to report, possibly more among elderly and men. Children whose parents were proxy responders over the internet exhibited the lowest false negative proportion, perhaps because reporting parents are particularly motivated or because parents might not be affected by illness at the same time as their children. Interestingly, men who did not respond quickly to the invitation showed an increased risk of producing invalid data. This could conceivably be construed as evidence against sending reminders to men who do not respond to the invitation and as an alternative either improve the invitation or apply oversampling in this group.

Although the cohort members were instructed to spontaneously report all new episodes of colds and fevers, the stated purpose was influenza surveillance. It is conceivable that cohort members limited their event-driven reports to what they interpreted as influenza and thus increased the false negative proportion.

In the 2008/2009 season we included, in addition to the randomly selected members of the general public, former cohort members who volunteered for a second season. While this may raise concerns about inflated validity and between-season similarities, analyses stratified according to recruitment mode (re-entered versus newly entered) convincingly allayed these concerns.

In the first season, but not in the second, we observed a moderate but non-significant increase in the false negative proportions over time. When controlling for available covariates in the first season, the probability of false negative reporting was 21% higher in week 7 than in week 1, but the ordinal variable was not significant. It appears that the underreporting was reasonably constant over time and between seasons. This was further corroborated by the fact that the level of underreporting among the first season’s cohort members – recruited around October 1^st^ and effectively having their validation study 3–6 months after entry into the cohort – was almost identical to that in the second season’s cohort, recruited in December and having their validation study 1–3 months after entry. Although we did not specifically study the validity in the very end of the seasons, the concordance of the estimated level of underreporting during the validation period and the estimate based on the end-of-follow-up questionnaire, covering the entire season, indirectly suggests that the underreporting remained fairly stable throughout the 9-month season. Therefore, adjustments for the underreporting should be possible. However, we have no evidence to support the generalizability of this constancy to other settings and/or other reported outcomes.

Notwithstanding underreporting and virtual absence of false positive event-driven reports, the weekly incidence proportions of ILI were almost one order of magnitude higher than the estimates for the population recalculated by the European Influenza Surveillance Scheme (EISS) based on Swedish sentinel data [22]. Furthermore the influenza epidemics 2007/2008 and 2008/2009 were quite dissimilar. The former was dominated by influenza B followed by influenza A H1, started slowly, and peaked late and rather unimpressively; and the latter was dominated by influenza A H3, with an earlier higher and more pronounced peak [23]–[24]. Still, the epidemic curves based on our event-driven reporting showed good correlation with curves obtained from the sentinel surveillance. The similarity provides further indirect support to the conclusion that the error in event-driven reporting is fairly constant within and between seasons. Similarities between data from other types of surveillance schemes based on self-reporting laymen and co-existing sentinel systems have previously been reported [25]–[27] but neither of these schemes relied on event-driven self-reporting nor were they population-based.

To demonstrate the feasibility, we corrected the observed weekly incidence proportions using our computed validity indices (Table 2). It should be noted that we used validity data concerning AURTI and fever to correct the ILI reporting. The adjusted ILI reporting gave an insight into the ILI disease burden on the community. However this estimate should not to be interpreted as the true influenza incidence rate since the applied ILI case definition may lack in sensitivity and specificity compared to laboratory testing for influenza [28]. More importantly, the exact magnitude of the error to be corrected for was uncertain due to the potential imperfections of the reference method discussed before.

Our surveillance data should be possible to use also for analytical studies of associations between background factors and risk of infections. With essentially no false positive reports, underreporting that is non-differential vis-à-vis studied background factors will not bias the risk-ratio estimate [29]. It will, however, underestimate the absolute magnitude of the risk difference, but predictably by a factor equal to the false negative proportion, again making corrections possible.

Whilst our findings regarding the validity of reports of common upper respiratory tract infections may have some bearing also on interpretation of reports of other outcomes in other cohort studies with passive follow-up, such generalizability can be no more than conjectural at present. It should also be emphasized that this study only addressed the validity of self-initiated, event-driven self-reporting of disease outcomes during prolonged periods of passive surveillance or follow-up, not the performance of the surveillance scheme per se. The evaluated validity constitutes an important piece of information, but the choice of surveillance scheme must also be governed by the primary purposes of the surveillance, and by local prerequisites such as the structure of health care and sickness insurance scheme, telecommunication infrastructure, as well as the educational level and motivation among the general public.

In conclusion, passive long-term surveillance through self-initiated, event-driven outcome reporting by members of representative general population cohorts underestimates the incidence of common upper respiratory tract infections. However, using the data provided in this validation study, incidence rates are potentially correctable, although with some uncertainty. Importantly, the generalizability to other populations/cultures needs to be confirmed. When launching similar surveillance schemes as ours, we find it advisable to also check the validity of the self-reports.

## Supporting Information

Table S1 Symptom questions asked when participants in the population-based surveillance system submit self-initiated event-driven self-reports. Below are the modified ECDC case definitions of acute upper respiratory tract infection, AURTI, and influenza-like illness, ILI applied in the surveillance (translated from Swedish). The questions are from the questionnaire for adults.
(DOCX)

Table S2 Characteristics of individuals selected for and included in the 2009 validation study, by mode of recruitment (re-entered from the 2008 surveillance cohort or newly entered).
(DOCX)

Table S3 Observed false negative proportion, false positive proportion and predictive values in the 2009 validation by age group and overall, stratified according to mode of recruitment (re-entered from the 2008 surveillance cohort or newly entered).
(DOCX)
