# Supplementary material for: The Validity of Self-Initiated, Event-Driven Infectious Disease Reporting in General Population Cohorts
Source: PLoS One. 2013 Apr 17;8(4):e61644. doi: 10.1371/journal.pone.0061644 (PMC3629155; doi:10.1371/journal.pone.0061644)
Supplement: Table S1 — Symptom questions asked when participants in the population-based surveillance system submit self-initiated event-driven self-reports. Below are the modified ECDC case definitions of acute upper respiratory tract infection, AURTI, and influenza-like illness, ILI applied in the surveillance (translated from Swedish). The questions are from the questionnaire for adults. (DOCX) [file pone.0061644.s001.docx]

| **The relative order of the questions** | | **The questions** |
| --- | --- | --- |
| **2007/2008** | **2008/2009** |  |
| 1 | 1 | Has it been less than eight days since you fell ill? (yes/no/ do not know or do not want to answer). |
| 2 | 2 | Did you fall ill suddenly, that is did you fall ill within a few hours? |
| 3 | 3 | Have you felt feverish at any time since you fell ill? |
| 4^a^ | 4^a^ | Was the temperature more than 38 degrees? |
| 5 | 5 | Have you coughed at any time since you fell ill? |
| 6 | 6 | Have you had muscle ache or other bodily ache at any time since you fell ill? |
| 7 | 10 | Have you been nauseous at any time since you fell ill? |
| 8 | 9 | Have you had headache at any time since you fell ill? |
| 9 | 8 | Have you had a sore throat at any time since you fell ill? |
| 10 | 11 | Have you experienced difficulties breathing/shortness of breath at any time since you fell ill? |
| 11 | 7 | Have you had a runny nose at any time since you fell ill? |
| 12 | 12 | Have you sought a doctor for your illness at any time since you fell ill? |
| 13^a^ | 13^a^ | Did the doctor say that it was an infection? |
| 14 | 14 | When did you fall ill? Answer by pressing the digit that corresponds to the number of days that have passed since you first noticed the illness. |
| **Case definitions** | | |
| AURTI | | Cough OR Sore throat OR Shortness of breath OR Coryza (runny nose) |
| ILI | | Sudden onset AND Cough OR Sore throat OR Shortness of breath |
|  | | AND Feverishness OR Headache OR Myalgia |

^a^ The question is only asked if the respondent answered yes to the previous question.
