# Supplementary material for: The Validity of Self-Initiated, Event-Driven Infectious Disease Reporting in General Population Cohorts
Source: PLoS One. 2013 Apr 17;8(4):e61644. doi: 10.1371/journal.pone.0061644 (PMC3629155; doi:10.1371/journal.pone.0061644)
Supplement: Table S2 — Characteristics of individuals selected for and included in the 2009 validation study, by mode of recruitment (re-entered from the 2008 surveillance cohort or newly entered). (DOCX) [file pone.0061644.s002.docx]

|  |  | **2009** | | | | | | | |
| --- | --- | --- | --- | --- | --- | --- | --- | --- | --- |
|  |  | **Selected (n=2514)** | | | | **Included in analysis^a^ (n=2134)** | | | |
|  |  | **Re-entered (n=1062)** | | **Newly entered (n=1452)** | | **Re-entered (n=974)** | | **Newly entered (n=1160)** | |
|  |  | **No.** | **%** | **No.** | **%** | **No.** | **%** | **No.** | **%** |
| Men |  | 420 | 40 | 654 | 45 | 375 | 39 | 497 | 43 |
| Age groups, years | ≤14 | 155 | 15 | 258 | 18 | 132 | 14 | 181 | 16 |
|  | 15-39 | 193 | 18 | 337 | 23 | 170 | 17 | 236 | 20 |
|  | 40-64 | 425 | 40 | 499 | 34 | 398 | 41 | 417 | 36 |
|  | 65-95 | 289 | 27 | 358 | 25 | 274 | 28 | 326 | 28 |
| Child ≤15 years |  | 167 | 16 | 281 | 19 | 144 | 15 | 195 | 17 |
| Subjects with at least one event-report |  | 470 | 44 | 523 | 36 | 438 | 45 | 472 | 41 |
| Total number of complete event-reports |  | 580 |  | 603 |  | 542 |  | 543 |  |

^a^ Included in the analysis were defined as selected individuals who returned at least one validation questionnaire with interpretable answers.
