# Supplementary material for: The Validity of Self-Initiated, Event-Driven Infectious Disease Reporting in General Population Cohorts
Source: PLoS One. 2013 Apr 17;8(4):e61644. doi: 10.1371/journal.pone.0061644 (PMC3629155; doi:10.1371/journal.pone.0061644)
Supplement: Table S3 — Observed false negative proportion, false positive proportion and predictive values in the 2009 validation by age group and overall, stratified according to mode of recruitment (re-entered from the 2008 surveillance cohort or newly entered). (DOCX) [file pone.0061644.s003.docx]

|  | **Age group** | **Re-entered** | | **Newly entered** | |
| --- | --- | --- | --- | --- | --- |
|  |  | % | 95% CI | % | 95% CI |
| False negative proportion | 0-14 | 65% | (38-86) | 50% | (32-68) |
|  | 15-39 | 67% | (35-90) | 52% | (30-74) |
|  | 40-64 | 48% | (26-70) | 64% | (46-79) |
|  | ≥65 | 73% | (39-94) | 71% | (48-89) |
|  | Total | 61% | (47-73) | 59% | (49-68) |
| False positive proportion | 0-14 | 2% | (0-7) | 1% | (0-5) |
|  | 15-39 | 0% | (0-3)^a^ | 2% | (0-5) |
|  | 40-64 | 0% | (0-1)^a^ | 1% | (0-2) |
|  | ≥65 | 0% | (0-2) | 0% | (0-2) |
|  | Total | 0% | (0-1) | 1% | (0-2) |
| Positive predictive value | 0-14 | 75% | (35-97) | 94% | (73-100) |
|  | 15-39 | 100% | (40-100)^a^ | 77% | (46-95) |
|  | 40-64 | 100% | (72-100)^a^ | 87% | (60-98) |
|  | ≥65 | 75% | (19-99) | 86% | (42-100) |
|  | Total | 89% | (71-98) | 87% | (75-95) |
| Negative predictive value | 0-14 | 90% | (83-95) | 87% | (80-92) |
|  | 15-39 | 94% | (89-97) | 94% | (89-97) |
|  | 40-64 | 97% | (95-99) | 93% | (90-96) |
|  | ≥65 | 97% | (93-98) | 94% | (91-97) |
|  | Total | 95% | (94-97) | 93% | (91-94) |

^a^One-sided, 97.5% confidence interval
